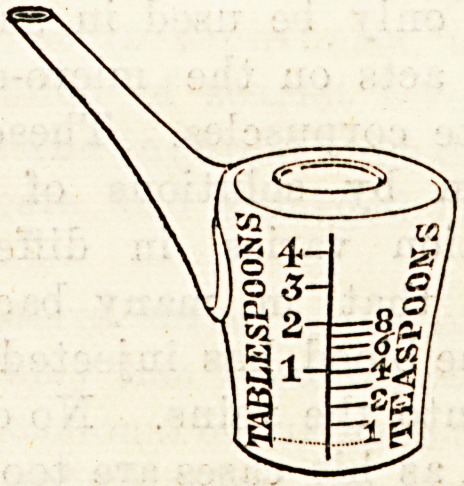# New Appliances and Things Medical

**Published:** 1894-03-03

**Authors:** 


### NEW APPLIANCES AND THINGS MEDICAL.

### ANTISEPTIC SOAPS.

(MESSRS. E. COOK AND CO., BOW, E.)

AT the invitation of Messrs. E. Cook and Co., we made a journey to their vast works at Bow with the express purpose of critically examining their methods of manufacture, and reporting thereon in the columns of this journal. Some time since we received samples of their various soaps, and have submitted some of those which are important from a medical point of view to a few crucial and searching tests. The manufacture of these medicated soaps forms but a very small fraction of the output of Messrs. Cook's works. Their energies are largely directed towards the manufacture of household and export soaps, which we saw in all stages of preparation, and immense quantities ready for transport to all parts of the world. But the soaps to which we propose in this article to call special attention are "the antiseptic," "the 10 per cent. carbolic," and "the hygienic tooth" soaps. It may surprise many of our readers to learn that all of these soaps are made from beef and mutton tallow and pure lard, quite as fresh as that supplied in the form of suet or lard by the West-end butchers to their West-end clientelle. The fats are purified, saponified by treatment with alkali, and subsequently made absolutely neutral by the removal of all free alkali. Before allowing the soap to cool and set, the final steps in the production of a correct consistency are carried out by one of the partners of the firm, as the process is one which entails skill and experience. The antiseptic, whether in the form of carbolic acid, biniodide of mercury, or the numerous constituents contained in the formula for the hygienic tooth soap, are added to the finished soap in its dry condition, so that neither the antiseptic properties of the drugs nor their due proportion is affected by a second boiling of the soap. The method of rubbing in the various ingredients is one of extreme ingenuity, and ensures the absolute homogenous consistence of the finished soap.

"The antiseptic" soap, which contain nearly 5 per cent. of the biniodide of mercury, is probably the most powerful germicide soap which can be safely entrusted in the hands of the public. Its value in cases of tinea tonsurans (ringworm) and other skin affection due to parasitic growth can probably not be overrated. If, however, Messrs. Cook are to make a commercial success of this preparation, they must reduce the size of these tablets, as in its present form one cake of their antiseptic soap is sufficient to treat a regiment. The 10 per cent. carbolic soap is what it claims to be, namely, a soap which "does" contain 10 per cent. of pure crystalline carbolic acid, and not 10 per cent. of the commercial acid, which represents about 5 per cent. of pure acid. We strongly recommend this soap to the notice of surgeons, and especially to those engaged in abdominal operations. The soap is agreeable to use, and does not roughen the hands to the same degree as does even a 1 in 40 solution of the acid.

The hygienic tooth soap is composed of a first-class dentifrice added to a basis of pure soap. It is decidedly pleasant on the gums, but is perhaps a trifle too sweet to the taste. It is supplied in neat little china capsules, very cleanly for the washing-stand, and convenient to carry in one's dressing-bag.

Those who wish to encourage home industries to the exclusion of American or foreign commodities of a like nature will do the country a service, and be the individual gainers, by patronising Messrs. Cook's most excellent specialities. We have no room to mention in detail the various forms of toilet soap supplied by the firm, but we know of no soap to equal their "Savon de Luxe" and "Reviere" soap (super fatted) for toilet purposes.

### THE HERCULES HORSE-ACTION SADDLE.

Messrs. Vigor and Co., 21, Baker Street, Portman Square, W.

The above firm have just taken out a patent for this saddle, and have submitted one of them to us for inspection. The object of the invention is to supply a substitute for horse exercise within the reach of moderate incomes. The invention consists of a saddle mounted upon a strong stand, and supported upon a central axis, which allows an up and down movement, which is resisted and limited by an arrangement of springs and checks. By means of movable clamps, which may be fixed in any position, the motion may be so altered so as very closely to resemble the actual movements of riding a horse at a trot or a gallop, the great difference being that in the case of the Hercules saddle the rider does the work of both man and beast. The amount of exercise which may thus be obtained in a few minutes would probably have to be expressed in "foot-tons." It is of decidedly a pleasant character, and brings into play almost exactly the same muscles as those employed in horse exercise. There is, of course, some degree of monotony in the motion, and it wants the element of fresh air and the exhilarating effect which attends the equestrian, but we regard it as a useful substitute for horse exercise. For persons above twenty-five years of age it is in many ways altogether preferable to other forms of gymnastics, and has the advantage of being really agreeable. It certainly gives the operator a good shaking, and brings strongly into play the abdominal muscles, a great desideratum to those of sluggish liver or atonic condition of bowel. The price of the machine varies from £7 to £22.

### A NEW MEDICINE GLASS.

(Messrs. Lynch and Co., 192, Aldersgate Street, E.C.)

Messrs. Lynch have sent us a medicine glass and measure which will assuredly find favour amongst nurses and such of the public as perforce are obliged to take medicine. Coates' Patent Measure, as the glass is called, resembles somewhat a small feeder made in glass, and the doses marked at the side. Medicine by this means can be administered to a very weak patient without causing fatigue, whilst iron or other drugs injurious to the teeth may be taken by the convalescent far more conveniently than through the medium of a tube, which is easily broken, and difficult to keep clean. The contrivance is a simple one, but it will be found an excellent addition to the conveniences of the sick room.

## Figures and Tables

**Figure f1:**
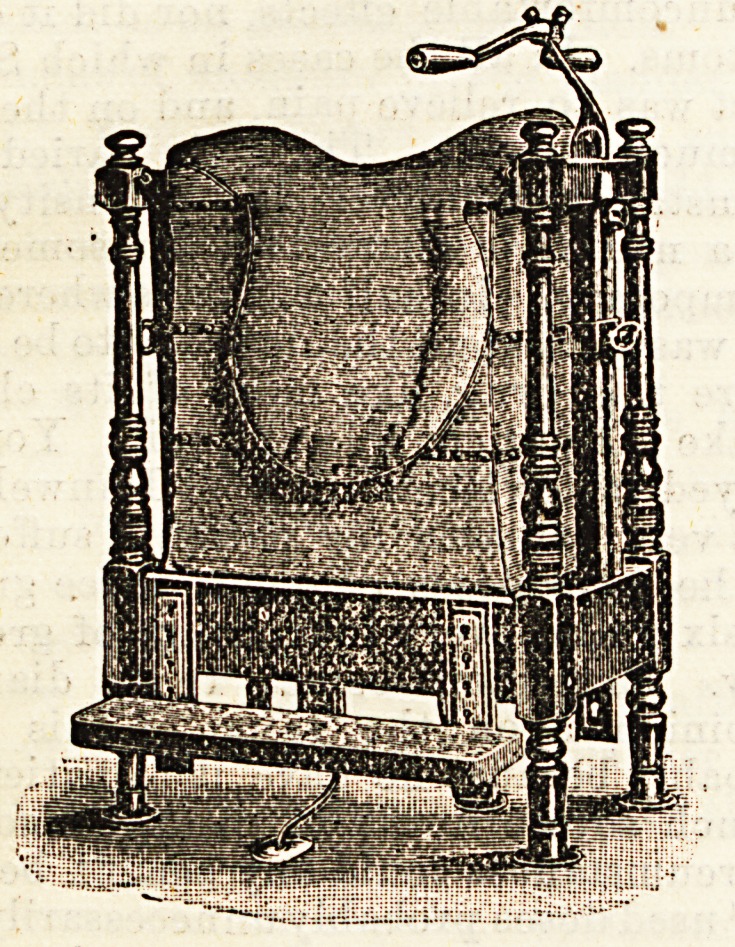


**Figure f2:**